# Just 2% of SARS-CoV-2−positive individuals carry 90% of the virus circulating in communities

**DOI:** 10.1073/pnas.2104547118

**Published:** 2021-05-10

**Authors:** Qing Yang, Tassa K. Saldi, Patrick K. Gonzales, Erika Lasda, Carolyn J. Decker, Kimngan L. Tat, Morgan R. Fink, Cole R. Hager, Jack C. Davis, Christopher D. Ozeroff, Denise Muhlrad, Stephen K. Clark, Will T. Fattor, Nicholas R. Meyerson, Camille L. Paige, Alison R. Gilchrist, Arturo Barbachano-Guerrero, Emma R. Worden-Sapper, Sharon S. Wu, Gloria R. Brisson, Matthew B. McQueen, Robin D. Dowell, Leslie Leinwand, Roy Parker, Sara L. Sawyer

**Affiliations:** ^a^BioFrontiers Institute, University of Colorado Boulder, Boulder, CO 80303;; ^b^Department of Molecular, Cellular, and Developmental Biology, University of Colorado Boulder, Boulder, CO 80303;; ^c^Department of Biochemistry, University of Colorado Boulder, Boulder, CO 80303;; ^d^HHMI, University of Colorado Boulder, Boulder, CO 80303;; ^e^Darwin Biosciences Inc., Boulder, CO 80303;; ^f^Interdisciplinary Quantitative Biology Program, University of Colorado Boulder, Boulder, CO 80303;; ^g^Wardenburg Health Center, University of Colorado Boulder, Boulder, CO 80303;; ^h^Department of Integrative Physiology, University of Colorado Boulder, Boulder, CO 80303;; ^i^Department of Computer Science, University of Colorado Boulder, Boulder, CO 80303

**Keywords:** viral load, SARS-CoV-2, transmission

## Abstract

We analyze data from the fall 2020 pandemic response efforts at the University of Colorado Boulder, where more than 72,500 saliva samples were tested for severe acute respiratory syndrome coronavirus 2 (SARS-CoV-2) using qRT-PCR. All samples were collected from individuals who reported no symptoms associated with COVID-19 on the day of collection. From these, 1,405 positive cases were identified. The distribution of viral loads within these asymptomatic individuals was indistinguishable from what has been previously observed in symptomatic individuals. Regardless of symptomatic status, ∼50% of individuals who test positive for SARS-CoV-2 seem to be in noninfectious phases of the disease, based on having low viral loads in a range from which live virus has rarely been isolated. We find that, at any given time, just 2% of individuals carry 90% of the virions circulating within communities, serving as viral “supercarriers” and possibly also superspreaders.

## Significance

We analyzed data from saliva-based COVID-19 screening deployed on the University of Colorado Boulder campus. Our dataset is unique in that all SARS-CoV-2−positive individuals reported no symptoms at the time of saliva collection, and therefore were infected but asymptomatic or presymptomatic. We found that 1) the distribution of viral loads observed in our asymptomatic college population was indistinguishable from what has been reported in hospitalized populations; 2) regardless of symptomatic status, approximately 50% of individuals who test positive for SARS-CoV-2 seem to be in noninfectious phases of the infection; and 3) just 2% of infected individuals carry 90% of the virions circulating within communities, serving as viral “supercarriers” and likely also superspreaders.

Severe acute respiratory syndrome coronavirus 2 (SARS-CoV-2) is a novel coronavirus that emerged into the human population in late 2019 (1), presumably from animal reservoirs (2, 3). During the ensuing world-wide pandemic, already more than 3 million lives have been lost due to the virus. Spread of SARS-CoV-2 has thus far been extremely difficult to contain. One key reason for this is that both presymptomatic and asymptomatic infected individuals can transmit the virus to others (4–13). Further, it is becoming clear that certain individuals play a key role in seeding superspreading events (14–17). Here, we analyzed data from a large university surveillance program. Viral loads were measured in saliva, which has proven to be an accessible and reliable biospecimen in which to identify carriers of this respiratory pathogen, and the most likely medium for SARS-CoV-2 transmission (18–20). Our dataset is unique in that all SARS-CoV-2−positive individuals reported no symptoms at the time of saliva collection, and therefore were infected but asymptomatic or presymptomatic. We find that the distribution of SARS-CoV-2 viral loads on our campus is indistinguishable from what has previously been observed in symptomatic and hospitalized individuals. Strikingly, these datasets demonstrate dramatic differences in viral levels between individuals, with a very small minority of the infected individuals harboring the vast majority of the infectious virions.

## Results

### The University of Colorado Boulder SARS-CoV-2 Screening Operation.

We analyzed data resulting from SARS-CoV-2 testing performed on the University of Colorado Boulder campus during the fall academic semester of 2020 (August 27 to December 11, 2020). Residents of dormitories were tested weekly, and several campus testing sites were in operation throughout the semester, offering testing for any campus affiliate. At the time of saliva collection, participants were asked to confirm that symptoms were not present; therefore, any infected persons identified through this surveillance testing were asymptomatic or presymptomatic at the time of saliva collection. It should be noted that all of the samples analyzed herein were collected before the B.1.1.7 (“U.K.”) SARS-CoV-2 variant, and subsequent major variants of concern, were first documented in the United States during the final weeks of 2020 and the beginning of 2021 (21).

During the fall 2020 semester, more than 72,500 saliva samples were screened for SARS-CoV-2. A qRT-PCR assay was used, with the template coming from the direct addition of saliva without RNA purification (22). Three TaqMan primer/probe sets were used in a multiplex reaction directed against two regions of the SARS-CoV-2 genome (CU-E and CU-N, where CU stands for the University of Colorado) and a host transcript (CU-RNaseP) as control. The multiplex reaction was used to create standard curves to convert Ct value (cycle threshold) of each primer set to viral load (virions per milliliter) in the original saliva sample (*SI Appendix*, Fig. S1*A*). To ensure the viral load quantification is accurate for samples with extremely low Ct values (i.e., extremely high viral loads), we performed serial dilution of three saliva samples with among the highest observed viral loads of the semester, and showed that Ct values scale linearly with the dilution factor (*SI Appendix*, Fig. S1*B*).

**Fig. 1.**
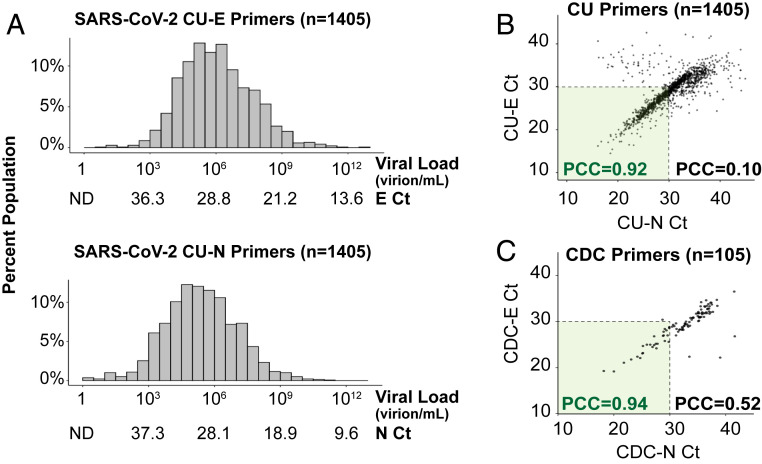
Saliva viral load distribution within our campus population. (*A*) Distributions are shown of the viral loads measured in the 1,405 positive samples identified on campus during the fall semester of 2020. Each histogram shows Ct values obtained using TaqMan primer/probe sets targeting either the E gene (“CU-E”) or the N gene (“CU-N”) of SARS-CoV-2. The horizontal axes are labeled with both the Ct values and the corresponding viral loads calculated from the standard curve for each primer set (*SI Appendix*, Fig. S1*A*). ND denotes no data, as the viral load is below the qRT-PCR detection limit. (*B*) The Ct values resulting from the two primer sets in *A* are highly correlated, especially in samples with high viral loads (Ct value lower than 30). Pearson correlation coefficients (PCC) are shown within and beyond the Ct = 30 arbitrary cutoff. (*C*) For 105 of the SARS-CoV-2−positive saliva samples, we ran qRT-PCR side by side with eight different primer sets commonly used in SARS-CoV-2 diagnostic tests (*SI Appendix*, Fig. S2). Here, we show the same analysis as in *B*, except with the CDC primers targeting the E and N genes (see *Methods*).

From over 72,500 saliva samples screened, 1,405 SARS-CoV-2−positive samples were identified. The vast majority of these positive samples were from unique individuals, because individuals with positive tests were directed into the health care system for further testing and care. The distribution of the Ct values of these 1,405 individuals, with each of the two primer sets used, is shown in Fig. 1*A*. Overall, the distribution of SARS-CoV-2 viral load fits under a log-normal distribution centered around the mean of 2.1 × 10^7^ virions per mL (median = 1.1 × 10^6^ virions per mL) for the CU-E primers or 5.9 × 10^6^ virions per mL (median = 2.5 × 10^5^ virions per mL) for the CU-N primers (*SI Appendix*, Fig. S3). The highest observed viral load was over 6 trillion (6.1 × 10^12^) virions per mL, which was only observed in one individual. It is remarkable to consider that this individual was on campus and reported no symptoms at our testing site. The lowest viral load detected was eight virions per milliliter. Thus, surveillance testing demonstrates an extremely wide variation in the viral load in infected but seemingly healthy (asymptomatic) individuals.

To verify that these viral load distributions were not influenced by the specific qRT-PCR primers used, we determined the agreement between the CU-N and CU-E primers with regard to the Ct values produced from samples. Different primer sets should be expected to produce slightly different Ct values on the same sample, due to differences in primer efficiencies and human pipetting error during reaction setup. Nonetheless, we find a tight correlation in samples with Ct values of <30 (Pearson correlation coefficient between CU-N and CU-E Ct values = 0.92), but this correlation breaks down in samples with higher Ct values (Pearson correlation coefficient between CU-N and CU-E Ct values = 0.10; Fig. 1*B*). At high Ct values (i.e., low viral loads), weaker correlation is likely a result of stochasticity in reverse transcription and/or in the initial rounds of PCR. This is supported by an in-depth analysis performed on 105 of the SARS-CoV-2−positive samples, where each sample was analyzed with eight different primer sets commonly used in SARS-CoV-2 diagnostic tests (Fig. 1*C* and *SI Appendix*, Fig. S2). We see tight congruence between Ct values generated with different primers on the same samples, especially at Ct values of <30. Overall, since the CU-E primer set demonstrated the highest consistency with other primer sets during this in-depth comparison (*SI Appendix*, Fig. S2), we used the Ct values resulting from this primer set to calculate saliva viral loads from this point forward.

### Populations Have Similar Viral Load Distributions Regardless of Symptomatic Status.

**Fig. 2.**
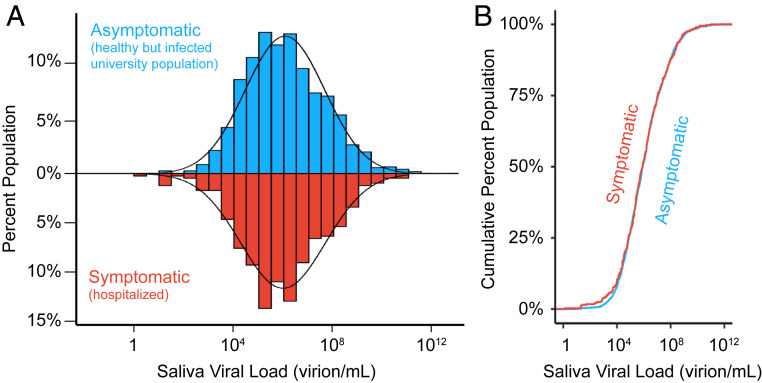
Viral load distributions are similar in asymptomatic and symptomatic populations. (*A*) A histogram of saliva viral loads in our asymptomatic campus population (*n* = 1,405, blue) compared to the same histogram of saliva viral loads from symptomatic (*n* = 404, red) individuals. The latter represents data compiled from the 10 studies in *SI Appendix*, Table S1. A log-normal probability density function is fitted onto the two distributions given the population mean and standard deviation. (*B*) Empirical cumulative distribution functions (ECDFs) of saliva viral load in the asymptomatic (*n* = 1,405, blue) and symptomatic (*n* = 404, red) populations. The similarity of the two ECDFs was assessed with the Kolmogorov−Smirnov test, which resulted in D statistic = 0.03, and *P* value = 0.97.

We next compared viral loads from individuals on our campus, who had no symptoms at the time of sample collection, to similar viral load measurements taken in saliva of symptomatic individuals. We examined published SARS-CoV-2 qRT-PCR datasets derived from studies of hospitalized (and therefore symptomatic) individuals. We specifically sought studies that assayed saliva and where viral loads were reported, since Ct values are laboratory and assay specific (23). We identified 404 data points that met such criteria, which we collated from the 10 studies listed in *SI Appendix*, Table S1. We note that our campus sampling likely represents earlier average time points in the course of infection than that of the hospital samples, which were mostly collected after symptom onset. Nonetheless, similar to the viral load distribution of the campus asymptomatic population (mean = 2.1 × 10^7^ virions per mL, median = 1.1 × 10^6^ virions per mL), the viral load in symptomatic patient saliva samples shows a log-normal distribution with a mean of 2.5 × 10^7^ virions per mL (median = 9.4 × 10^5^ virions per mL) and varied from very high viral loads (9.5 × 10^10^ virions per mL) to viral loads near the limit of detection (1.3 virions per mL) (Fig. 2*A* and *SI Appendix*, Fig. S3). We next plotted the cumulative distribution of viral load in both populations (Fig. 2*B*). This comparison really represents two extremes: One group is mostly hospitalized, while the other group represents a mostly young and healthy (but infected) college population. Yet, the distributions are extremely similar (two-sided two-sample Kolmogorov−Smirnov test, D statistic = 0.03, *P* value = 0.97; Fig. 2*B*). Therefore, individuals have similar distributions of saliva viral load regardless of symptomatic status, as has also been observed in studies of viral load in anterior nasal or nasopharyngeal swabs (24–29).

### A Small Subset of Individuals Carries Most of the Circulating Virions.

**Fig. 3.**
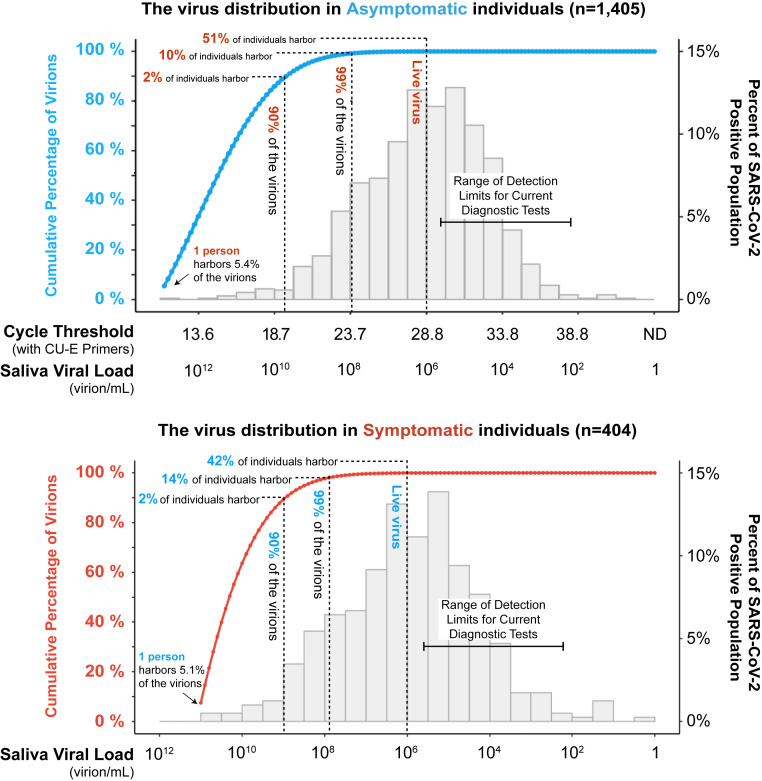
A small percentage of individuals are viral supercarriers. The histograms shown (right *y* axes) are the same as were shown in Fig. 2. Starting from the left of each histogram (i.e., those individuals with the highest viral loads), we calculated the accumulative percentage of total virions as a function of saliva viral load based on the probability density function of the distribution (blue and red lines, and left *y* axes). In both asymptomatic (blue line) and symptomatic populations (red line), the portion of population that harbors 90% and 99% of the circulating virus is highlighted by the dashed lines. We estimate that only around 50% (51% and 42% in the panels shown) of individuals who test positive for the virus actually harbor infectious virions, based on the observation that live virus has rarely been isolated from samples with viral loads of <10^6^ virions per mL (28, 30–35). For context, the range of detection limits of common SARS-CoV-2 diagnostic testing paradigms (qRT-PCR, antigen testing, and reverse transcription loop-mediated isothermal amplification) are shown. All testing paradigms will capture virtually all infectious individuals and virions, in presymptomatic and symptomatic populations alike. Limits of detection are taken from refs. 50 to 52.

We next analyzed how virus is distributed between individuals within populations. By summing the viral load across individuals based on the interpolated probability density function representing each population, starting with those with the highest viral loads, we find that just 2% of individuals harbor 90% of the circulating virions (Fig. 3). This is true in both the university (i.e., asymptomatic) and hospitalized (i.e., symptomatic) populations. Further, 99% of community-circulating virions are accounted for by just 10% of the asymptomatic and 14% of the symptomatic population. In both asymptomatic and symptomatic populations, one single individual with the highest saliva viral load carried more than 5% of the total circulating virions. On the other hand, all individuals with saliva viral loads lower than 10^6^ virions per mL combined (representing ∼50% of the infected individuals) harbor less than 0.02% of the virions in both populations. This can be understood because Ct is a linear representation of logarithmic increases in viral load, so that the viral load increases exponentially as the Ct value decreases (*SI Appendix*, Fig. S1). Thus, there is a highly asymmetric distribution of viruses within both populations, with just a small number of people carrying the vast majority of the virus. It remains unknown whether these are special individuals capable of harboring extraordinarily high viral loads, or whether many infected individuals pass through a very short time period of extremely high viral load (see further discussion below). Irrespective of mechanism, it is nevertheless true that, at any given moment in time, a small number of people are harboring the vast majority of virions.

Infectious virions have rarely been isolated from clinical samples of individuals with viral load less than 10^6^ virions per mL (28, 30–35). One hypothesis is that people in this low range of viral load may simply be shedding viral genomes from damaged tissue that is undergoing repair, and, for this reason, they may not pose a substantial risk of infecting others. Our distributions suggest that approximately half of the people who test positive may not be infectious to others (Fig. 3), based on this line of reasoning.

## Discussion

An important finding herein is that the vast majority of circulating virions in communities are found within the bodies of a small number of individuals. These findings corroborate similar trends observed elsewhere (14–17, 25). Although it remains to be seen exactly how transmission probability relates to viral load, a strong implication is that these individuals who are viral supercarriers may also be superspreaders. Higher viral loads have been shown to increase the probability of transmission to others in China (36), in Spain (37), and between pairs of roommates on our university campus (38). A higher rate of spread by viral supercarriers would be consistent with recent contact tracing analyses suggesting that 80 to 90% of infections are caused by 10 to 20% of infected individuals (14–17). A higher rate of spread by viral supercarriers would also be consistent with the surprisingly low transmission rates being reported between roommates (38), schoolmates (39, 40), and household members (41), which could be explained if only a small fraction of infected individuals have high enough viral loads to facilitate active transmission.

One potential explanation for the differences in viral loads between individuals is that individuals were simply tested at different stages of otherwise similar viral infections. However, longitudinal analyses of individual infections show that peak viral loads vary dramatically between individuals (42–44). Thus, the parsimonious explanation is that individuals produce different levels of virus. Whether this is due to variation in the immune response, variation in host factors supporting virus replication like ACE2, the specific viral variant infecting, or initial infection site or dose remains to be determined (45–48). To look at this further, we compared the viral load distributions analyzed herein to a theoretical normal distribution using quantile−quantile plots (*SI Appendix*, Fig. S3). The data deviate from the normal distribution at the extreme ends, including in the part of the population with the highest viral loads. This is consistent with the hypothesis that a small percentage of individuals represent a unique population with different capacity for infection than the rest of the population.

The concentration of a majority of the virus in a small fraction of the population at a given time is a critical observation with actionable conclusions. Community screening to identify viral supercarriers within presymptomatic and asymptomatic stages of disease will be important, since these individuals will continue to sustain and drive the epidemic if not located. Finding viral supercarriers will have a disproportionately large impact on curbing new COVID-19 infections, yet individuals without symptoms don’t tend to seek out testing, so screening will need to target healthy populations. Modeling approaches show that one of the most important factors in screening for SARS-CoV-2 will be the speed with which infected people receive their test results (also referred to as turnaround time) (49). The longer it takes for people to receive their results, the more time goes by where they might unwittingly infect others. Therefore, it is imperative that we find virus supercarriers, and inform them of their infection status in a way that is fast, easy, and accessible. Although detection limits vary between current monitoring and diagnostic paradigms, all are more than capable of finding the majority of infected individuals and the vast majority of circulating virions (Fig. 3) (50–52).

## Methods

### Collection of University Samples.

For sample collection conducted at our university, individuals were asked to fill out a questionnaire (https://www.colorado.edu/daily-health-form) to confirm that they did not present any symptoms consistent with COVID-19, and to collect no less than 0.5 mL of saliva into a 5-mL screw-top collection tube. Saliva samples were heated at 95 °C for 30 min on site to inactivate the viral particles for safer handling, and then placed on ice or at 4 °C before being transported to the testing laboratory for qRT-PCR analysis on the same day.

### Saliva qRT-PCR Used for Screening Saliva Samples on the University of Colorado Boulder Campus.

For qRT-PCR analysis, the university testing team transferred 75 μL of saliva into one well of a 96-well plate where each well had been preloaded with 75 μL of 2× Tris/borate/ethylenediaminetetraacetic acid (TBE) buffer supplemented with 1% Tween-20. Of this diluted sample, 5 μL was then added to one well of a separate 96-well plate where each well had been preloaded with 15 μL of reaction mix composed of TaqPath 1-Step Multiplex Master Mix (Thermo Fisher A28523), nuclease-free water, and triplex primer mix consisting of CU-E, CU-N, and CU-RNaseP primer and probe sets (Table 1; conditions changed slightly during the semester). The reagents were mixed, spun down, and loaded onto a Bio-Rad CFX96 or CFX384 qPCR machine. The qRT-PCR was run using the standard mode, consisting of a hold stage (25 °C for 2 min, 50 °C for 15 min, and 95 °C for 2 min) followed by 44 cycles of a PCR stage (95 °C for 3 s, 55 °C for 30 s, with a 1.6 °C/s ramp-up and ramp-down rate). Ct values from all campus testing efforts were communicated to us as deidentified data.

**Table 1.** The qRT-PCR TaqMan primer/probe sets used for university screening and focused analysis

Assay name	Primer/probe set target and designation	Primer or probe name	1× concentration, nM	Sequence (5′ to 3′)*
CU	SARS-CoV-2 E gene “CU-E”	E_Sarbeco_F1 (IDT 10006888)	400	ACA​GGT​ACG​TTA​ATA​GTT​AAT​AGC​GT
		E_Sarbeco_R2(IDT 10006890)	400	ATA​TTG​CAG​CAG​TAC​GCA​CAC​A
		E_Sarbeco_P(IDT Custom)	200	TexRd-ACACTAGCCATCCTTACTGCGCTTCG- IAbRQSp
	SARS-CoV-2 N gene “CU-N”	nCOV_N1_F (IDT 10006830)	500	GAC​CCC​AAA​ATC​AGC​GAA​AT
		nCOV_N1_R (IDT 10006831)	500	TCT​GGT​TAC​TGC​CAG​TTG​AAT​CTG
		nCOV_N1_P (IDT Custom)	250	HEX-ACCCCGCAT-ZEN-TACGTTTGGTGGACC-IABkFQ
	Human RNase P“CU-RNaseP”	RNaseP_F (IDT 10006836)	50	AGA​TTT​GGA​CCT​GCG​AGC​G
		RNaseP_R (IDT 10006837)	50	GAGCGGCTGTCTCCACAA GT
		RNase_P_P (IDT 10006838)	50	FAM-TTCTGACCT-ZEN-GAAGGCTCTGCGCG-IABkFQ
CDC	SARS-CoV-2 E gene “CDC-E”	E_Sarbeco_F1 (IDT 10006888)	400	ACA​GGT​ACG​TTA​ATA​GTT​AAT​AGC​GT
		E_Sarbeco_R2 (IDT 10006890)	400	ATA​TTG​CAG​CAG​TAC​GCA​CAC​A
		E_Sarbeco_P (IDT 10006893)	200	FAM-ACACTAGCCATCCTTACTGCGCTTCG- IABkFQ
	SARS-CoV-2 N gene “CDC-N”	nCOV_N2_F (IDT 10006833)	600	TTA​CAA​ACA​TTG​GCC​GCA​AA
		nCOV_N2_R (IDT 10006834)	600	GCGCGACATTCCGAAGAA
		nCOV_N2_P (IDT 10007049)	300	SUN- ACA​ATT​TGC​CCC​CAG​CGC​TTC​AG - IABkFQ
	Human RNase P “CDC-RNaseP”	RNaseP_F (IDT 10006836)	50	AGA​TTT​GGA​CCT​GCG​AGC​G
		RNaseP_R (IDT 10006837)	50	GAGCGGCTGTCTCCACAA GT
		RNase_P_P (IDT 10007062)	50	ATTO647-TTCTGACCTGAAGGCTCTGCGCG - IABkFQ
SalivaDirect (20)	SARS-CoV-2 N gene “SalivaDirect-N”	nCOV_N1_F (IDT 10006830)	400	GAC​CCC​AAA​ATC​AGC​GAA​AT
		nCOV_N1_R (IDT 10006831)	400	TCT​GGT​TAC​TGC​CAG​TTG​AAT​CTG
		nCOV_N1_P (IDT 10006832)	200	FAM-ACCCCGCATTACGTTTGGTGGACC-IABkFQ
	Human RNase P “SalivaDirect-RNaseP”	RNaseP_F (IDT 10006836)	150	AGA​TTT​GGA​CCT​GCG​AGC​G
		RNaseP_R (IDT 10006837)	150	GAGCGGCTGTCTCCACAA GT
		RNase_P_P (IDT 10007062)	200	ATTO647-TTCTGACCTGAAGGCTCTGCGCG - IABkFQ

* Explanation for some of the TaqMan fluorophores and quenchers used: IAbRQSp, Iowa Black Dark Quenchers RQ; IABkFQ, Iowa Black Dark Quenchers FQ; ZEN, internal quencher; TexRd, Texas Red; HEX, Hexachloro-fluorescein; FAM, fluorescein.

### Focused Analysis of 105 SARS-CoV-2−Positive Samples.

For a smaller subset of 105 samples, as described herein, we did a side-by-side comparison of three different qRT-PCR multiplex assays commonly used in SARS-CoV-2 diagnostics. We thawed 105 frozen, deidentified saliva samples which had previously tested positive for SARS-CoV-2 in the campus screening operation and performed all of the following qRT-PCR analyses side by side on the day of sample thawing.

First, 25 μL of thawed, previously heat-treated saliva was transferred into one well of a 96-well plate where each well had been preloaded with 25 μL of 2× TBE buffer supplemented with 1% Tween-20. Next, 5 μL of the diluted sample was added to separate 96-well plates where each well had been preloaded with 15 μL of reaction mix composed of TaqPath 1-Step Multiplex Master Mix (Thermo Fisher A28523), nuclease-free water, and US Centers for Disease Control’s (CDC) triplex primer mix or CU triplex primer mix (Table 1). The reagents were mixed, spun down, and loaded onto a Bio-Rad CFX96 qPCR machine. The qRT-PCR was run using the standard mode, consisting of a hold stage (25 °C for 2 min, 50 °C for 15 min, and 95 °C for 2 min) followed by 44 cycles of a PCR stage (95 °C for 3 s, 55 °C for 30 s, with a 1.6 °C/s ramp-up and ramp-down rate). Each plate also contained two wells of negative control template (5 μL of nuclease-free water diluted 1:1 with 2× TBE supplemented with 1% Tween-20) and two wells of positive control template (5 μL of synthetic SARS-CoV-2 RNA [Twist Biosciences 102024] diluted to 1,000 genome copies per μL, and 5 μL of total human reference RNA [Agilent 750500] diluted to 10 ng/μL in nuclease-free water).

We also performed the SalivaDirect TaqMan qRT-PCR analysis (20) on each of these samples; 75 μL of each saliva specimen was combined with 9.4 μL of Proteinase K (20 mg/mL, New England Biolabs, P8107S). Samples were incubated at ambient temperature for 15 min and then heated to 95 °C for 5 min to inactivate the Proteinase K. Next, 5 μL of saliva was used as template in a 20-μL reaction that also contained 1× TaqPath 1-Step Multiplex Master Mix, nuclease-free water, and primer and probe sets at concentrations described below. The qRT-PCR was run on the BioRad CFX96 qPCR machine using the same program described for the CU assays (20).

### Ethics Statements.

Viral load data on university participants were deidentified and aggregated from the University of Colorado Boulder operational screening for SARS-CoV-2. This activity does not meet the definition of human subject research described in the United States Health and Human Services 45 Code of Federal Regulations Part 46. Deidentified saliva samples (*n* = 105) used for the cross-comparison of primers were collected under protocol 20-0662, approved by the University of Colorado Boulder Institutional Review Board.

## Supplementary Material

Supplementary File

## Data Availability

All study data are included in the article and *SI Appendix*.
