# Supplementary material for: Just 2% of SARS-CoV-2−positive individuals carry 90% of the virus circulating in communities
Source: Proc Natl Acad Sci U S A. 2021 May 10;118(21):e2104547118. doi: 10.1073/pnas.2104547118 (PMC8166196; doi:10.1073/pnas.2104547118)
Supplement: Supplementary File [file pnas.2104547118.sapp.pdf]

SUPPORTING INFORMATION

Supplementary Table S1. Studies from which viral loads in symptomatic individuals were derived*

STUDY	HOSPITAL, COUNTRY	GENDER (MALE/FEMALE)	AGE	METHOD
**TO ET AL.[1]**	Princess Margaret Hospital and Queen Mary Hospital, Hong Kong	13/10	37-75	Posterior oropharyngeal saliva followed by RT-qPCR
**TO ET AL.[2]**	Princess Margaret Hospital and Queen Mary Hospital, Hong Kong	7/5	37-75	Posterior oropharyngeal saliva followed by RT-qPCR
**ZHANG ET AL.[3]**	Wuhan Pulmonary Hospital, China	NA	NA	Saliva from oral swab followed by RT-qPCR
**HANEGE ET AL.[4]**	Goztepe Education and Research Hospital, Turkey	11/18	26-70	Self-collected saliva followed by RT-qPCR
**PROCOP ET AL.[5]**	Cleveland Clinic, USA	25/14	18-82	Self-collected saliva followed by RT-qPCR
**ZHENG ET AL.[6]**	First Affiliated Hospital, College of Medicine, China	58/38	44-64	Self-collected saliva after deep cough followed by RT-qPCR
**YOON ET AL.[7]**	Korea University Guro Hospital, Korea	0/2	46-65	Self-collected saliva followed by RT-qPCR
**WYLLIE ET AL.[8]**	Yale New Haven Hospital, USA	NA	NA	Self-collected saliva followed by RT-qPCR
**YOKOTA ET AL.[9]**	Hokkaido University Hospital, Japan	25/17	27-93	Self-collected saliva followed by RT-qPCR
**ZHU ET AL.[10]**	Central Hospital of Xiangtan, China	16/16	34-54	Self-collected saliva followed by RT-qPCR

* All studies indicated that saliva samples were self-collected from COVID-19 patients at the indicated locations. In all cases, the authors reported virus concentrations in the original saliva sample.

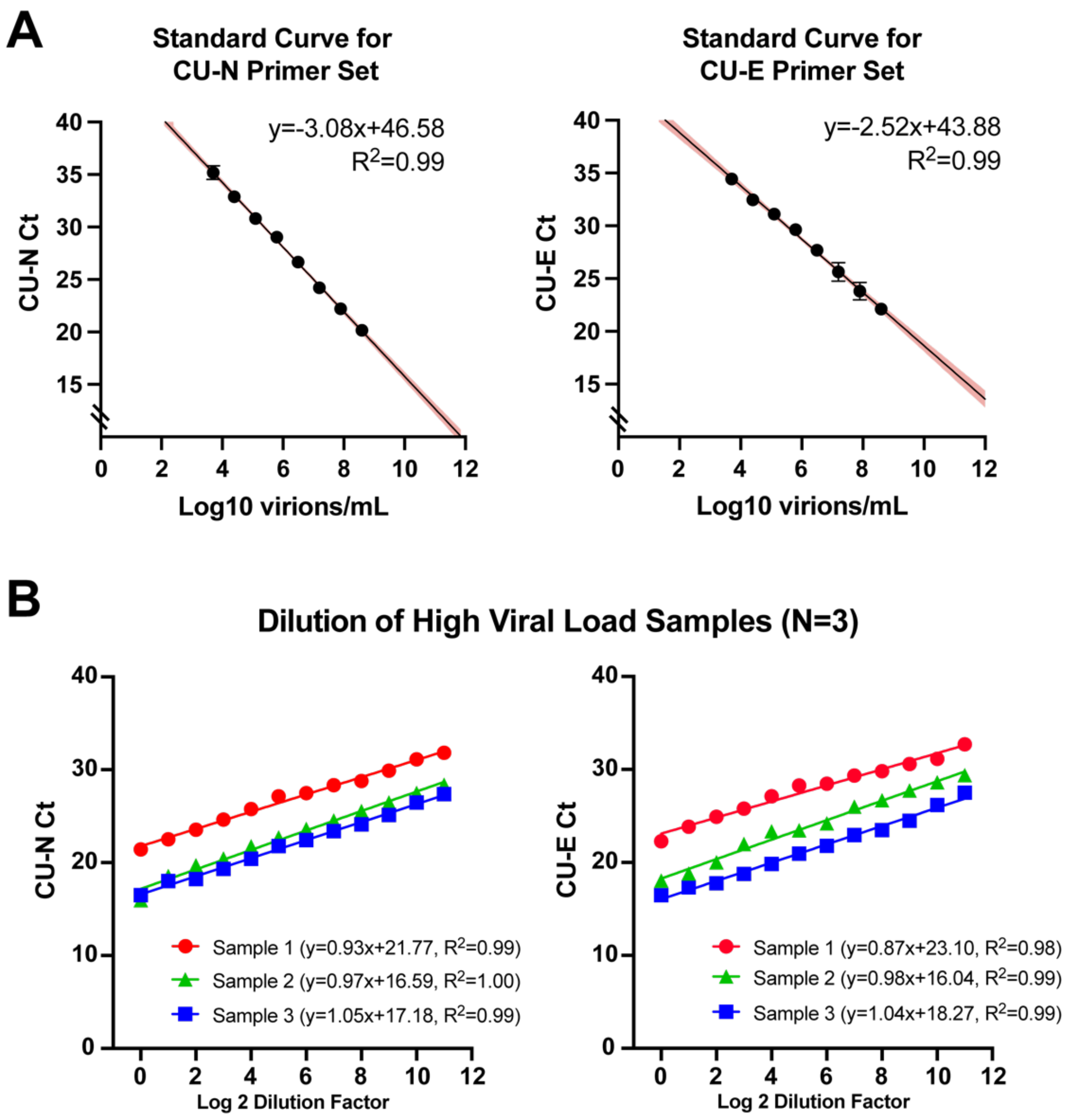

Supplementary Figure S1. Examination of the linearity of the RT-qPCR assay over the range of viral loads observed in this study. (A) Standard curves were created for the primer sets used in this study. Gamma-irradiated SARS-CoV-2 virions (BEI Resources NR-52287) were spiked into healthy saliva samples from three different individuals to reach 4×10^8 virions/mL and incubated for 30 minutes at 95ºC. Samples were then serially diluted 1:5 using heat-treated healthy saliva from the same individuals as the diluent, yielding the indicated final concentrations (X-axis). Samples were then subjected to the multiplex RT-qPCR reaction described in the method. The standard curve for each primer set was generated by linear regression analysis of the triplicate experiment and is illustrated with 95% confidence interval (R-squared>0.99 for both standard curves). **(B)** We performed serial dilution of three of the saliva samples with amongst the highest observed viral loads of the semester (8.1×10^8 – 1.2×10^{11} virions/mL) to determine the linear range of the RT-qPCR assay. Saliva was incubated for 30 minutes at 95ºC, then diluted 1:2 in series using heat-treated healthy saliva as the diluent. Linear regression was performed on the dilution series to show that the Ct values scale linearly with dilution factors (R-squared > 0.98 for all dilution curves).

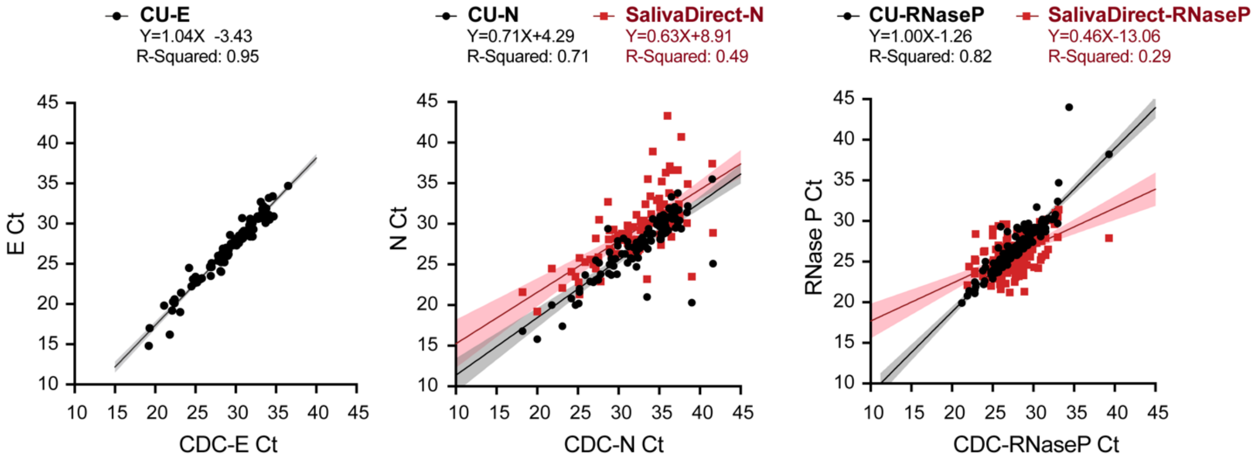

Supplementary Figure S2. Correlation of Ct values between different primer sets used to quantify saliva viral load. Using 105 SARS-CoV-2 positive saliva samples, we examined the Ct values obtained with different RT-qPCR multiplex assays and compared them via correlation analysis. For 105 virus-positive saliva samples, 8 different Ct values were generated all in one day from each sample, in a side-by-side direct analysis of the performance of each primer set. Ct values from the Centers for Disease Control primers (CDC-E, CDC-N or CDC-RNaseP) are reported on the X-axes. On the Y-axes are plotted the corresponding Ct values resulted from our university screening primers (CU-E, CU-N or CU-RNaseP) and primer sets used in the SalivaDirect [11] test (SalivaDirect-N and SalivaDirect-RNase P) primer sets.

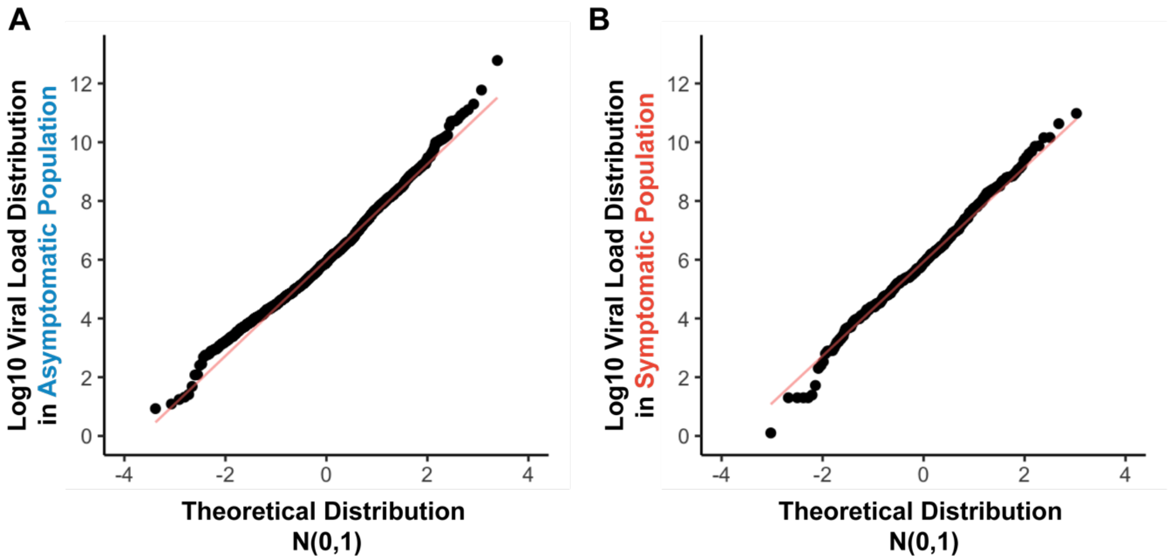

Supplementary Figure S3. The observed viral loads follow a normal distribution, except at the extreme ends. We compared the viral load data in each population (Y axis) to the theoretical standard normal distribution (X-axis) using a quantile-quantile plot. The points indicate the empirical quantiles of the datapoints, while the diagonal line (red) indicates the expected quantiles under normal distribution. The data deviates from the Gaussian distribution at the extreme ends, which likely represents individuals with either very high or very low viral loads.

SI References

1. To KK-W, Tsang OT-Y, Yip CC-Y, et al. Consistent detection of 2019 novel coronavirus in saliva. Clin Infect Dis Official Publ Infect Dis Soc Am **2020**;

2. To KK-W, Tsang OT-Y, Leung W-S, et al. Temporal profiles of viral load in posterior oropharyngeal saliva samples and serum antibody responses during infection by SARS-CoV-2: an observational cohort study. Lancet Infect Dis **2020**; 20:565–574.

3. Zhang W, Du R-H, Li B, et al. Molecular and serological investigation of 2019-nCoV infected patients: implication of multiple shedding routes. Emerg Microbes Infec **2020**; 9:386–389.

4. Hanege FM, Kocoglu E, Kalcioglu MT, et al. SARS-CoV-2 Presence in the Saliva, Tears, and Cerumen of COVID-19 Patients. Laryngoscope **2020**;

5. Procop GW, Shrestha NK, Vogel S, et al. A Direct Comparison of Enhanced Saliva to Nasopharyngeal Swab for the Detection of SARS-CoV-2 in Symptomatic Patients. J Clin Microbiol **2020**; 58.

6. Zheng S, Fan J, Yu F, et al. Viral load dynamics and disease severity in patients infected with SARS-CoV-2 in Zhejiang province, China, January-March 2020: retrospective cohort study. Bmj **2020**; 369:m1443.

7. Yoon JG, Yoon J, Song JY, et al. Clinical Significance of a High SARS-CoV-2 Viral Load in the Saliva. J Korean Med Sci **2020**; 35:e195.

8. Wyllie AL, Fournier J, Casanovas-Massana A, et al. Saliva or Nasopharyngeal Swab Specimens for Detection of SARS-CoV-2. New Engl J Med **2020**; 383:1283–1286.

9. Yokota I, Hattori T, Shane PY, et al. Equivalent SARS-CoV-2 viral loads between nasopharyngeal swab and saliva in symptomatic patients. medRxiv **2020**;

10. Zhu J, Guo J, Xu Y, Chen X. Viral dynamics of SARS-CoV-2 in saliva from infected patients. J Infection **2020**; 81:e48–e50;

11. Vogels CBF, Watkins AE, Harden CA, et al. SalivaDirect: A simplified and flexible platform to enhance SARS-CoV-2 testing capacity. Med **2020**.
